# A224 CHARACTERIZATION OF VERY-EARLY-ONSET INFLAMMATORY BOWEL DISEASE IN CHILDREN ampersand:003C6 YEARS OF AGE: A SINGLE CENTER EXPERIENCE

**DOI:** 10.1093/jcag/gwad061.224

**Published:** 2024-02-14

**Authors:** K S Alsaedi, A Sant'Anna, N Ahmed

**Affiliations:** Pediatric Gastroenterology, McGill University, Montreal, QC, Canada; Pediatric Gastroenterology, McGill University, Montreal, QC, Canada; Pediatric Gastroenterology, McGill University, Montreal, QC, Canada

## Abstract

**Background:**

Very-early-onset inflammatory bowel disease (VEO-IBD), diagnosed before the age of six years old, presents a distinct subtype of pediatric inflammatory bowel disease (IBD). This chronic inflammatory condition is believed to result from an abnormal immune response triggered by environmental factors including the gut microbiota, in genetically susceptible individuals.

**Aims:**

This study aims to analyze the clinical characteristics and management of VEO-IBD patients in a tertiary medical center, evaluating disease presentation, localization and treatment strategies.

**Methods:**

A retrospective descriptive study was conducted of 28 VEO-IBD patients diagnosed between January 2001 and January 2019 at our institution. Data was abstracted including patient demographics, clinical features, laboratory and histological parameters, treatment, and disease classification based on the revised Porto Criteria.

**Results:**

Among the 28 patients, Crohn’s disease (CD) was the predominant diagnosis (89%) with predominantly colonic involvement. Thiry-two percent of patients had isolated colitis whereas the upper gastrointestinal (UGI) tract was involved in 57% of the cohort, approximately one third had involvement of the small bowel. The most common presenting symptoms were rectal bleeding and diarrhea, both occurred in 65% of the patients. Patients were followed for a mean duration of 115 months. Despite prior assumptions of its severity, VEO-IBD patients in this cohort responded well to conventional treatments in which 64% were induced with steroid therapy, 25% received biologics and 35% received immunomodulators. Surgical interventions were relatively infrequent. Only 2 patients out the 28 VEO-IBD patients underwent surgery for refractory colitis, one of them in the 1^st^ 2 years of life. There were limited genetic testing results which made it difficult to know our incidence of monogenic IBD. However, only 5 of our patients had full genetic workup done in other centers for research purposes with 1 patient being diagnosed with monogenic IBD consistent IPEX syndrome.

**Conclusions:**

This study highlights the unique clinical profile of VEO-IBD, with CD being the prevalent diagnosis in this age group in our population. Our cohort did not have a more severe phenotype, emphasizing that there is significant variation within this group Understanding the predictors of response is needed in individual patients with VEO-IBD is vital for tailored management and improved outcomes in this young population.

**Funding Agencies:**

None

